# Spectroscopic and electrochemical study of interactions between DNA and different salts of 1,4-dihydropyridine AV-153

**DOI:** 10.7717/peerj.10061

**Published:** 2020-11-10

**Authors:** Elina Leonova, Karlis Shvirksts, Vitalijs Borisovs, Edgars Smelovs, Jelizaveta Sokolovska, Egils Bisenieks, Gunars Duburs, Mara Grube, Nikolajs Sjakste

**Affiliations:** 1 Faculty of Medicine, University of Latvia, Riga, Latvia; 2 Institute of Microbiology and Biotechnology, University of Latvia, Riga, Latvia; 3 Latvian Institute of Organic Synthesis, Riga, Latvia

**Keywords:** 1,4-dihydropyridines, DNA binding, AV-153 salts, G-quadruplexes, Spectrofluorimetry, Fourier-transformed infrared spectroscopy, Circular dichroism, Circular voltammetry

## Abstract

1,4-dihydropyridines (1,4-DHP) possess important biochemical and pharmacological properties, including antimutagenic and DNA-binding activity. The latter activity was first described for water-soluble 1,4-DHP with carboxylic group in position 4, the sodium salt of the 1,4-DHP derivative AV-153 among others. Some data show the modification of physicochemical properties and biological activities of organic compounds by metal ions that form the salts. We demonstrated the different affinity to DNA and DNA-protecting capacity of AV-153 salts, depending on the salt-forming ion (Na, K, Li, Rb, Ca, Mg). This study aimed to use different approaches to collate data on the DNA-binding mode of AV-153-Na and five other AV-153 salts. All the AV-153 salts in this study quenched the ethidium bromide and DNA complex fluorescence, which points to an intercalation binding mode. For some of them, the intercalation binding was confirmed using cyclic voltammetry and circular dichroism spectroscopy. It was shown that in vitro all AV-153 salts can interact with four DNA bases. The FTIR spectroscopy data showed the interaction of AV-153 salts with both DNA bases and phosphate groups. A preference for base interaction was observed as the AV-153 salts interacted mostly with G and C bases. However, the highest differences were detected in the spectral region assigned to phosphate groups, which might indicate either conformational changes of DNA molecule (B form to A or H form) or partial denaturation of the molecule. According to the UV/VIS spectroscopy data, the salts also interact with the human telomere repeat, both in guanine quadruplex (G4) and single-stranded form; Na and K salts manifested higher affinity to G4, Li and Rb –to single-stranded DNA.

## Introduction

Synthetic derivatives of 1,4-dihydropyridine (1,4-DHPs) possess various pharmacological properties, including antimutagenic and radioprotective effects. This study focused on a series of salts of the 1,4-DHP derivative AV-153. It was shown that AV-153-Na salt possesses antimutagenic activity, enhances the DNA repair, can protect DNA against genotoxic agents, and bind DNA (Buraka et al., 2014; Leonova et al., 2018; Ryabokon et al., 2005; Ryabokon et al., 2008; Ryabokon et al., 2009). The compound also stimulates cell growth, manifests anti-apoptotic activity, and possesses antioxidant properties (Milkovic et al., 2018). Different salts of the compound were recently synthesized and subjected to investigation. It is well-known that the formation of salt from a cyclic organic compound and metal causes a perturbation in the electronic system of the molecule. Alkali metals disturb the electronic structure of aromatic compounds. Metals also change the biological activity of organic compounds, including isonicotinates and nicotinic acid salts (Koczoń et al., 2005; Lewandowski, Kalinowska & Lewandowska, 2005), which are structurally close to 1,4-DHP. We showed in a recent study that metal ions modify the ability of the AV-153 to bind DNA and protect DNA in living cells against peroxynitrite or Tat-induced oxidative stress (Leonova et al., 2019). When administered to rats with experimental diabetes, AV-153-Na and AV-153-Ca produced different effects on the DNA repair-related and proteasomal genes (Dišlere, 2019). The metal ions remain complexed with the compound after dissociation, their modifying action on the AV-153 salt effects were produced in this dissociated state. The pKs of the salts ranged from pH 4.24 to 4.52, but all the experiments were performed at neutral pH (Leonova et al., 2019). These data encouraged us to conduct further studies of the interactions of AV-153 salts with DNA and proteins. We applied a set of physico-chemical methods to assess the impact of molecular modifications on the DNA binding mode. Interactions between the AV-153 salts and DNA were studied with cyclic voltammetry. The possible interaction mechanism was evaluated by means of EtBr displacement assay, circular dichroism, and infrared spectroscopy. We also tested the affinity of the AV-153 salts to telomeric repeats after taking the great interest in G-quadruplex-targeting drugs and recent reports on the possible application of the 1,4-DHP as G-quadruplex sensors into account (Aghaei et al., 2017).

## Materials and Methods

### Chemicals

**Figure 1:**
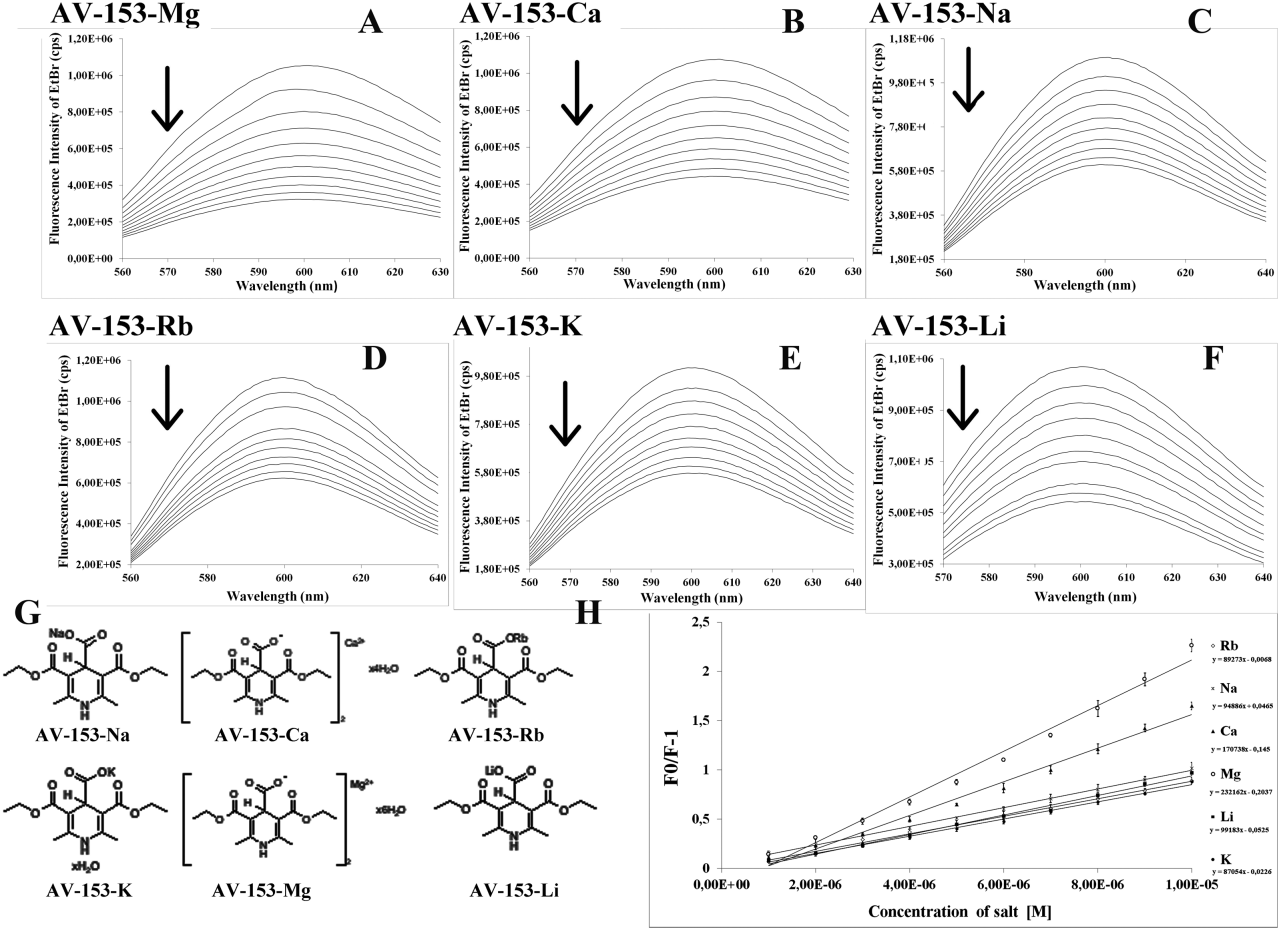
Spectrofluorimetric ethidium bromide displacement assay. (A–F) Changes in the spectra of EtBr-DNA complex induced by AV-153-Mg, AV-153-Ca, AV-153-Na, AV-153-Rb, AV-153-Rb, AV-153-Li, respectively. Six spectra out of 25 obtained are shown. (G) Formulas of the AV-153 salts. (H) Stern-Volmer plots.

Different AV-153 salts (3,5-bis-ethoxycarbonyl-2,6-dimethyl-1,4- dihydropyridine-4-carboxylate) were synthesized in the Laboratory of Membrane Active Compounds at the Latvian Institute of Organic Synthesis. The structure of the AV-153 salts are shown in Fig. 1. The synthesis of the 2,6-dimethyl-3.5-bis(ethoxycarbonyl)-1,4-dihydroisonicotinic acid was performed as reported (Duburs & Uldrikis, 1969). Salts of monovalent metals (Na, Li, K, Rb) were prepared following the same procedure. At the final stage of the synthesis, the saturated water solution of the appropriate hydroxide was added to the hot 2,6-dimethyl-3,5-bis(ethoxycarbonyl)-1,4-dihydroisonicotinic acid solution in ethanol. Salts of divalent metals (Ca and Mg) were prepared by transmetallation reaction to the sodium salt solution with the appropriate metal chloride in a water solution at room temperature. The obtained salts were dried in vacuo on phosphorus pentoxide and were fully characterized by ^1^H NMR, LC/MS and elemental analysis. The purity of the salts determined by HPLC exceeded 95%. Tris base, ethidium bromide (EtBr), calf thymus DNA (ct-DNA), human serum albumin (HSA), Na_2_EDTA, and inorganic salts were purchased from Sigma-Aldrich (Taufkirchen, Germany).

### Cyclic voltammetry

For the voltammetric experiments we used an EcoChemie Autolab PGSTAT 302T potentiostat/galvanostat (Utrecht, The Netherlands) and the electrochemical software package Nova 2.0. We used a three-electrode system: a two mm-sized Pt disk working electrode, an Ag/AgCl reference electrode (3 M KCl) and a Pt wire counter electrode purchased from Metrohm Co (Herisau, Switzerland). Voltammograms of the 5 mM AV-153 salt solution in 0.1 M Tris–HCl (pH = 7.4) were first registered. After that, 10 µM of DNA was added and the measurements were repeated. This was repeated twice or more. Scan rate was 100 mV/s. Electrodes were washed with double-distilled water before each measurement. Experiments were performed at 25 °C. Samples were deoxygenized with oxygen-free nitrogen gas.

The binding constant was determined using the equation:

\begin{eqnarray*}\log \nolimits \left( \frac{1}{DNA} \right) =\log \nolimits \left( K \right) +\log \nolimits \left[ {I}_{\mathrm{free}}/({I}_{\mathrm{free}}-{I}_{\mathrm{bond}}) \right] \end{eqnarray*}

where *K* is the apparent binding constant; *I*_free_ is the peak current of free compound; and *I*_bond_ is the peak current of compound in the presence of DNA (Feng, Li & Jiang, 1997).

The number of binding sites was determined using the equation:

\begin{eqnarray*} \frac{I-{I}_{DNA}}{{I}_{DNA}} = \frac{K \left[ DNA \right] }{2s} \end{eqnarray*}

where *I* is the peak current of a compound in the absence of DNA; A, IDNA is the peak current of a compound in the presence of DNA; A, K is the binding constant of compound-DNA complex; [DNA] is the DNA concentration, mol/L; s is the size of the binding site (bp) (Aslanoglu, 2006). The number of electrons (*n*) was calculated using the equation:

\begin{eqnarray*}{E}_{p}- \frac{{E}_{p}}{2} =47.7~\mathrm{mV }/\mathrm{\alpha }n \end{eqnarray*}

where *Ep* is the peak compound potential; mV; *Ep*/2 is the half-wave compound potential; mV, α –the assuming value = 0.539; n—number of electrons (Wang et al., 2011).

### Fluorescence spectroscopic measurements

Spectrofluorimetry was performed using a Fluoromax-3 (Horiba JOBIN YVON, China).

To study the interaction of 1,4-DHP with the DNA-EtBr complex by means of the spectrofluorimetry, the calf thymus DNA (74.8 µM) and ethidium bromide (1.26 µM) were diluted in 5 mM Tris HCl; 50 mM NaCl at pH 7.4. Spectra were recorded in a 1-cm cuvette (2 ml) at room temperature. The AV-153 salts were added by 8 µl aliquots of the 2.5 mM solution. The solution was mixed thoroughly and kept for 5 min to equilibrate before the fluorescence measurement. Interactions of the tested compounds with DNA caused displacement of EtBr from the complex with DNA. The fluorescence was recorded at 109,600 nm using an indirect excitation wavelength of EtBr at 260 nm (Geall & Blagbrough, 2000). The linear Stern-Volmer equation, in which 1,4-DHPs were the quenchers, was applied for calculation of the quenching constants:

\begin{eqnarray*} \frac{{I}_{0}}{I} =1+{K}_{sv} \left[ Q \right] \end{eqnarray*}

Where *I*_0_ and *I* represent the fluorescence intensities in the absence and presence of quenchers, respectively; *K*_*sv*_ is a linear Stern-Volmer constant; and *Q* is the quencher concentration. *K*_*sv*_ values were evaluated from the slope of the plot (Geethanjali et al., 2015). Linearity of the plots was tested by calculation of the correlation coefficient (Gong & Zhu, 2013; Wahba, El-Enany & Belal, 2015).

### Circular dichroism spectroscopy

CD spectra were recorded on a Chirascan CS/3D spectrometer (Applied Photophysics, Surrey, UK), DNA and compound binding measurements were done in 10 mM HEPES buffer, and pH 7.4 in a quartz cell of 10 mm path-length at room temperature. The CD spectra of ct-DNA and G4 structure were recorded in 220–320 nm range, and that of the HSA in a 200–260 nm range. The parameters for all spectra were as follows: scan rate (200 nm min^−1^), averaging time (0.125 s), bandwidth (1 nm) and one recorded spectrum is the average of four scans. We carried out titrations in the DNA region by adding increasing amounts of AV-153 salts (10 µM at each step) to a 50 µM DNA solution. Titrations in the induced CD compound region were performed by adding DNA (62.5 µM at each step) to a 500 µM AV-153-Na solution.

***UV/VIS spectroscopic measurements*** were applied for a study of the AV-153 salts interaction with bases (Sadeghi et al., 2016) and oligonucleotides corresponding to human telomere repeat sequence. The spectra were recorded with a Perkin Elmer Lambda 25 UV/VIS spectrophotometer. Spectra of a 25 µM solution of the tested compound in 50 mM NaCl and 5 mM Tris HCl at pH 7.4.were taken in a one cm quartz cell (2 ml) in the absence of bases and after adding 10 µM of bases.

In the case of human telomere repeats the titration was performed in 10 mM sodium phosphate buffer, 0.3 mM EDTA, 100 mM NaCl, pH 7.2, 50 nM of oligonucleotide were added each time.

The binding constants were calculated using the formula:

\begin{eqnarray*} \frac{1}{{A}_{0}-A} = \frac{1}{{A}_{0}} + \frac{1}{K\times {A}_{0}\times {c}_{DNA}} \end{eqnarray*}

Where *A*_0_ is absorption of the free substance; *A* is absorption in the presence of DNA; and *C*_*DNA*_ is the DNA concentration (Buraka et al., 2014).

### Fourier transform infrared (FTIR) spectroscopy

For the FTIR analyses AV-153-Li and AV-153-K were dissolved in distilled water (H2O), but AV-153-Ca, AV153-Mg, and AV-153-Rb were dissolved in dimethyl sulfoxide (DMSO). Aliquots containing from 5 to 10 µL of 7.5 mM DNA solutions, AV-153 salts or a mixture of DNA and the salts in a ratio of 1:5 were dried at T<50 °C on a 384-well silicone plate. The FTIR absorption spectra were recorded on a VERTEX 70 coupled with an HTS-XT microplate reader extension (BRUKER, Germany) over a 4,000–6,000 cm^−1^ range with a four cm^−1^ resolution, with 64 scans co-added. The baseline was corrected using the rubber band method, and CO_2_ bands were excluded. Spectra with absorption limits between 0.25 and 0.80 were used for data analyses in accordance with the Lambert-Bouger-Beer law, that is, the concentration of a component should be proportional to the intensity of the absorption band. The data were processed using OPUS 6.5 software.

### Interaction with single-stranded and guanine quadruplex forming oligonucleotides

Oligonucleotide corresponding to human telomere repeat (5′-AGGGTTAGGGTTAGGGTTAGGG-3′) was ordered in Metabion. To induce the formation of the antiparallel G4, the oligonucleotide was heated at 90° and allowed to slowly cool down for 1 h at room temperature and then for 3 d in 10 mM sodium phosphate buffer, 0.3 mM EDTA, 100 mM NaCl, pH 7.2 at +4 °C in a 10 mm path-length quartz cell at room temperature. The G4 formation was checked with circular dichroism spectroscopy (CD). To observe the interactions of AV-153 salts with the oligos, 5 µM of the oligonucleotide were titrated in 10 mM sodium phosphate buffer, 0.3 mM EDTA, 100 mM NaCl, pH 7.2 with 10 µM of AV-153 salt at each step, CD spectra were recorded after each stabilization of the solution (5 min) in a 220–320 nm range at room temperature. In a parallel binding of the oligo in G4 and single-stranded form (before the 3rd day of incubation in the presence of salt) was assayed with UV/VIS spectroscopy, as described above. A solution of AV-153 salts of monovalent metals (25 µM) in the buffer was titrated with either G4 or single-strand oligonucleotides, 50 nM each time.

### Statistics

All measurements were performed in triplicate. Numerical data are presented as mean ± S.E.M.

## Results

### Electrochemical study of the interactions between AV-53 salts and DNA

**Figure 2:**
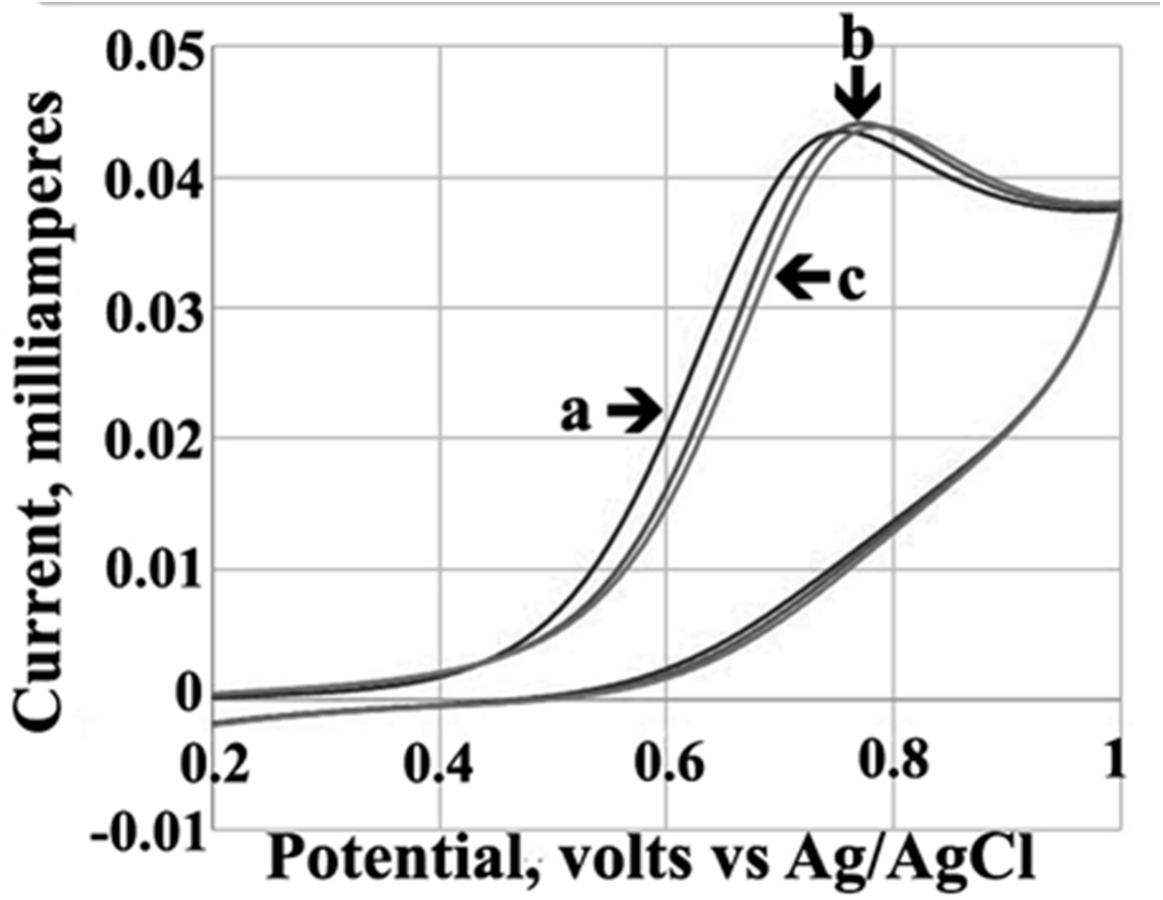
Cyclic voltammograms. Solution of 5 mM AV-153-K in 0.1 M Tris-HCl buffer (pH 7.4) without DNA (a) and in the presence of 10 µM (b) and 20 µM of DNA (c).

Cyclic voltammograms of 5 mM AV-153-K in the absence and presence of various DNA concentrations in 0.1 M Tris–HCl buffer, pH = 7.4 are shown in Fig. 2. The peak potential shifted to a more positive value (0.018 V) in the presence of DNA. A slight increase in the peak current upon the addition of increasing concentrations of DNA was also observed. The compound exhibited a single well defined anodic peak, which corresponds to the oxidation of the dihydropyridine ring (Augustyniak et al., 2010). No peak was observed in a reverse scan, indicating that the oxidation of the compound is an irreversible process. The peak current (*Ip*) of the oxidation wave AV-153-K was proportional to the square root of the scan (v^1∕2^). The cyclic voltammograms of other AV-153 salts were similar (not shown). The differences in the binding constants of the AV-153 salts were calculated using cyclic voltammetry data followed the same trend as affinity to DNA formerly determined spectrofluorimetrically (Leonova et al., 2019) (see Table 1).

### Fluorescent intercalator displacement assay

To assess the mode of the DNA interaction with the AV-153 salts we have performed a fluorescent intercalator displacement assay. The method is based on the quenching of the enhanced fluorescence of the DNA-EtBr complex by a second ligand (Ghosh et al., 2010). As presented in Fig. 1A, all AV-153 salts quenched the EtBr fluorescence up to 70%, indicating that the compounds competed with EtBr for DNA intercalation sites. The Stern-Volmer quenching constant calculations indicated that Ca and Mg salts displaced the EtBr from intercalation sites more intensively than Na, Li and K salts, and Rb salt produced the weakest effect. (Fig. 1B, Table 2).

### Circular dichroism

**Figure 3:**
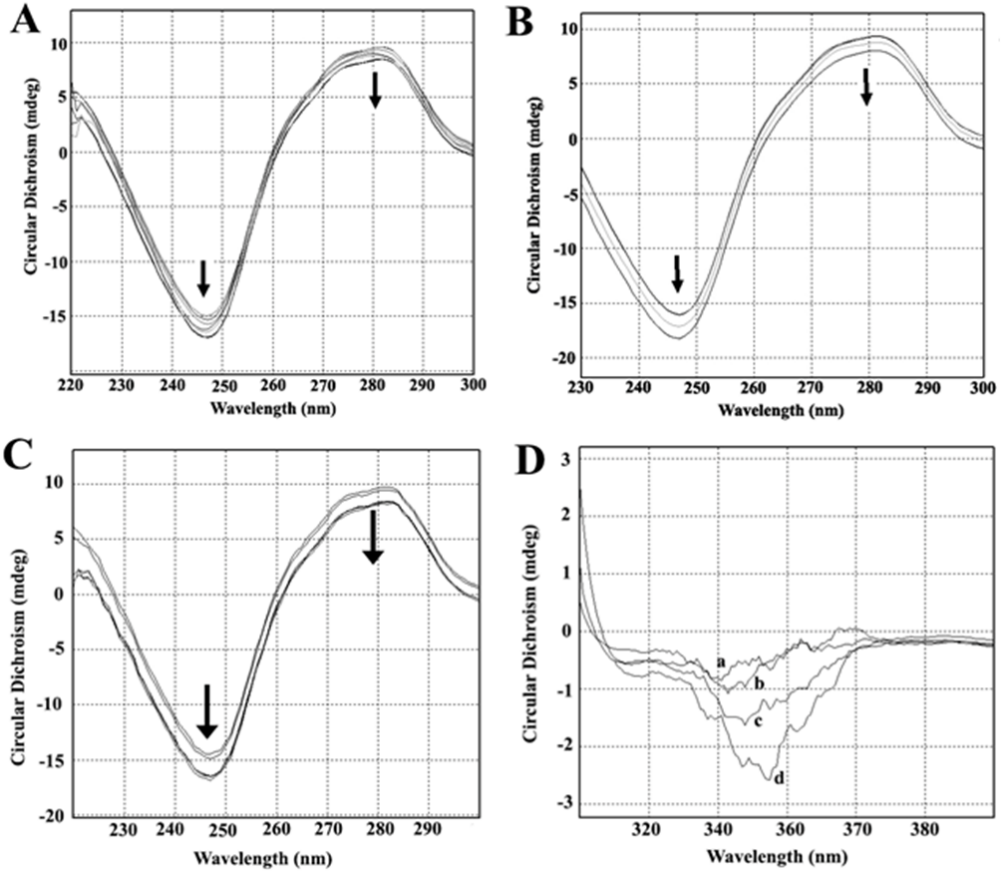
Circular dichroism experiments. Circular dichroism spectra of ct- DNA in absence and presence of (A) AV-153-Ca, (B) AV-153-Rb and (C) AV-153-Na. AV-153 salts concentration was increased by 10 µM at each step up to 40 µM. DNA concentration was 50 µM. Measurements were performed in 10 mM HEPES buffer. (D) Induced circular dichroism spectra of AV-153-Na (500 µM) in presence of 62.5 µM (A), 125 µM (B); 250 µM (C) and 500 µM (D) of DNA. Measurements were performed in HEPES buffer.

Circular dichroism spectra of ct-DNA in the presence of increasing concentrations of AV-153-Rb and AV-153-Ca are shown in Fig. 3. The DNA manifested a negative band at 245 nm due to helicity and a positive band at 270 nm because of base stacking which is characteristic of the B form of DNA. Adding AV-153 salts to DNA increased the negative band intensity and decreased the positive band intensity. A 2 nm red shift of crossover point was also observed. These data clearly indicate interactions of the compound with DNA, although changes in spectra are not typical for any binding mode.

### Interaction with bases

**Figure 4:**
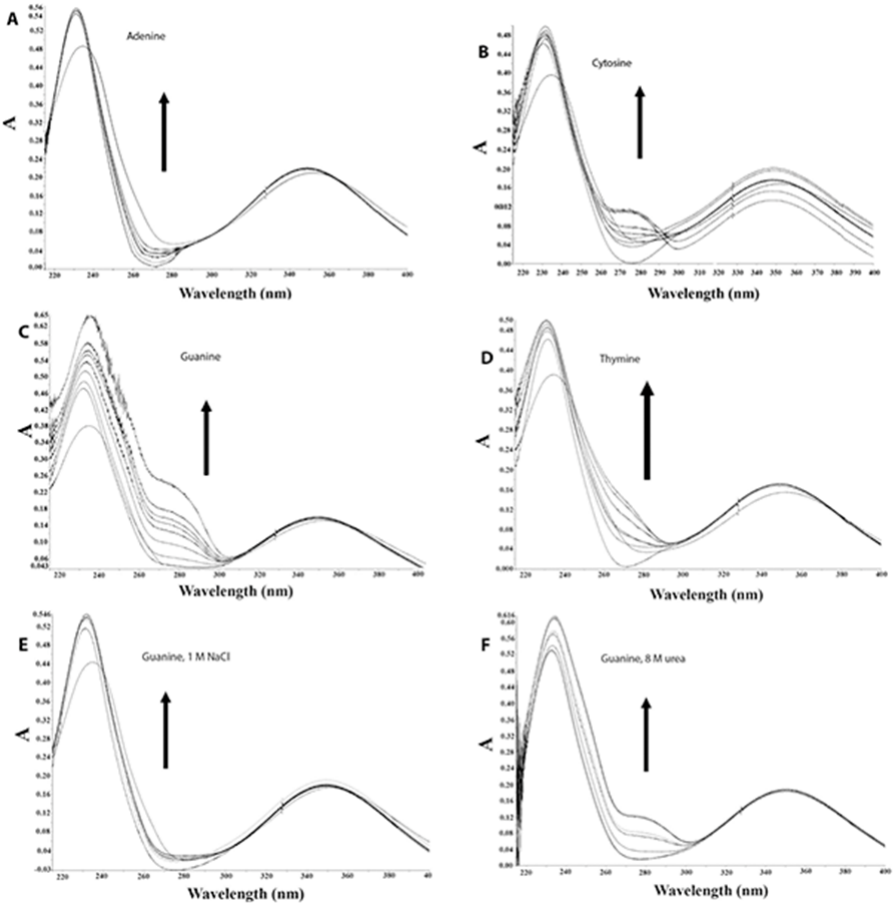
AV-153-Na absorption spectra in absence and presence of bases in different solutions. Concentrations of bases were increased for 10 µM with each titration. (A) Adenine, (B) Cytosine, (C, E, F) Guanine, (D) Thymine. (A–D) in 5 mM Tris HCl, pH 7.4, 50 mM NaCl; (E) 1 M NaCl; (F) 8 M urea.

The above results indicate that the binding between AV-153-Na and DNA partly occurs via intercalation. The influences of DNA bases, C, G, A and T, on the UV/VIS absorption spectra of AV-153 were used to evaluate the possible base-specificity of binding (Fig. 4, Table 3). The absorption intensity gradually increased with an increase in the concentration of all the four bases. Affinity to G, C and T is greater than that to A. The results indicate that AV-153-Na can interact with the four types of bases, with somewhat different affinities. Titration was performed in solutions of 1M NaCl and 8M urea to evaluate the role of ionic and hydrogen bonds in AV-153-Na interactions with bases. During this process, the media affinity of AV-153-Na to bases was weakened, especially for G, and the shape of the spectra was also changed. However, interactions were not abolished.

**Table 1:** Affinity of the AV-153 salts to DNA determined by spectrofluorimetry and DNA binding parameters determined by cyclic voltammetry.

Compound	Protein binding constant	DNA binding constant, cyclic voltammetry	Number of electrons (n)	Number of binding sites DNA (s)	Number of binding sites protein
AV-153-Na	3.9 × 10^5^				0.93
AV-153-Mg	4.3 × 10 ^5^	3.44 × 10^4^	0.52	4.87	0.93
AV-153-Ca	4.9 × 10^5^	2.755 × 10^5^	0.64	7.37	1.03
AV-153-K	5.2 × 10 ^5^	2.28 × 10^4^	0.71	2.60	1.12
AV-153-Li	1.08 × 10^6^	7.20 × 10^4^	0.54	5.60	2.25
AV-153-Rb	4.8 × 10^5^	6.83 × 10^4^	0.84	4.40	1.02

### FTIR spectroscopy of AV-153 salts and their complexes with DNA

**Figure 5:**
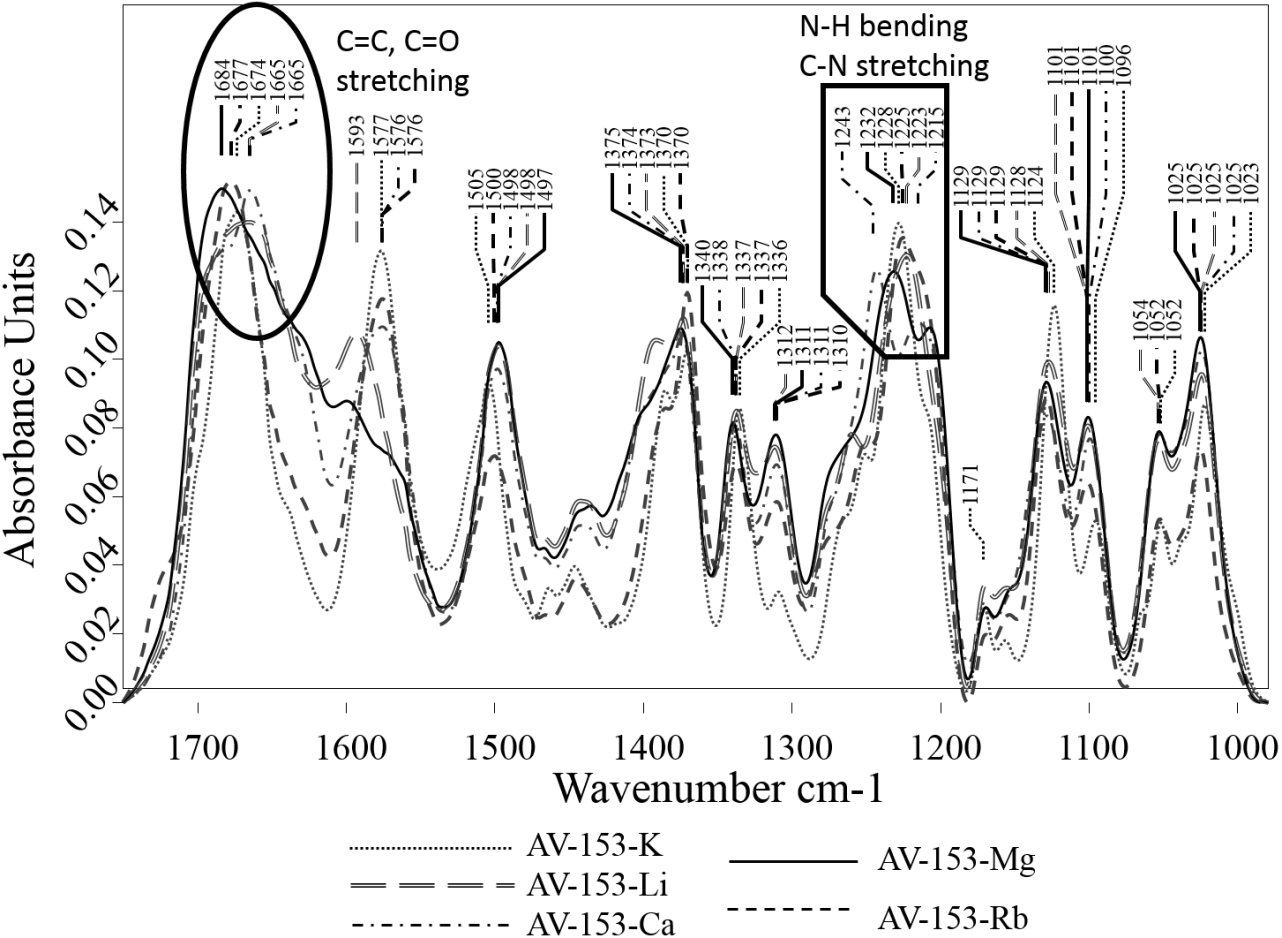
Vector normalized FTIR spectra of AV-153 salts. Major variations of C=O/C=C and N-H/C-N bands at ∼1,675 and ∼1,234 cm^−1^, respectively are shown.

The FTIR spectra of AV-153 salts showed several strong absorption bands (Fig. 5). In the spectra of all AV-153 salts, the most intensive were broad absorption bands with maximums in the wavenumber 1,220–1,243 cm^−1^ range, assigned to N-H bending and C-N stretching vibrations (Zhang et al., 2013), and 1,665–1,684 cm^−1^, assigned to CO, C=O, C=N stretching vibrations (Varsanyi, 1974). The shape of the absorption bands in the wavenumber region of 1,570–1,670 cm^−1^was quite different: (i) two sharp separate bands in spectra of AV-153 salt with K, Rb, Ca; (ii) two partly overlapping bands at 1,593 and 1,666 cm^−1^ in AV-153- Li; (iii) a broad strongly overlapping band with maximum at 1,684 cm^−1^. All the AV-153 salt spectra showed absorption bands at ∼1,025, ∼1,045, ∼1,101 and ∼1,128 cm^−1^, with variable mutual intensities. The broad and strong absorption bands in the 900–1,200 cm^−1^ region were assigned to the stretching vibrations of C-O, C-C bonds and C-O, C-C, C-O-O deformation vibrations, though many other functional groups have bands in this region as well (Naumann & Meyers, 2000). Also, a separate strong absorption band in the 1,497–1,505 cm^−1^ region with slightly varying intensity (the lowest in AV-153- Rb) and the band at ∼1,498 cm^−1^ in spectra of Li, CA and Mg AV-153 salts while at 1,500 and 1,505 cm^−1^ in spectra of AV-153 salts with Rb and K correspondingly.

**Table 2:** The ability of the AV-153 salts to extrude the EtBr from intercalation sites.

Compound	Ksv, M^−1^	Fluorescence decrease, % (+100 µM of compound)	Correlation coefficient
AV-153-Na	9.4 × 10^4^	44	.99
AV-153-Ca	1.7 × 10^5^	60	.99
AV-153-Mg	2.3 × 10^5^	65	.99
AV-153-Li	9.9 × 10^4^	50	.99
AV-153-K	8.7 × 10^4^	42	.99
AV-153-Rb	8.9 × 10^4^	45	.99

The evaluation of AV-153 salt spectral band intensities and shape showed major variations in the bands at ∼1,675 cm^−1^, assigned to the C=O and/or C=C bonds and ∼1,234 cm^−1^, assigned to the N-H and/or C-N bonds. These variations indicate the different metals binding with the AV-153.

**Figure 6:**
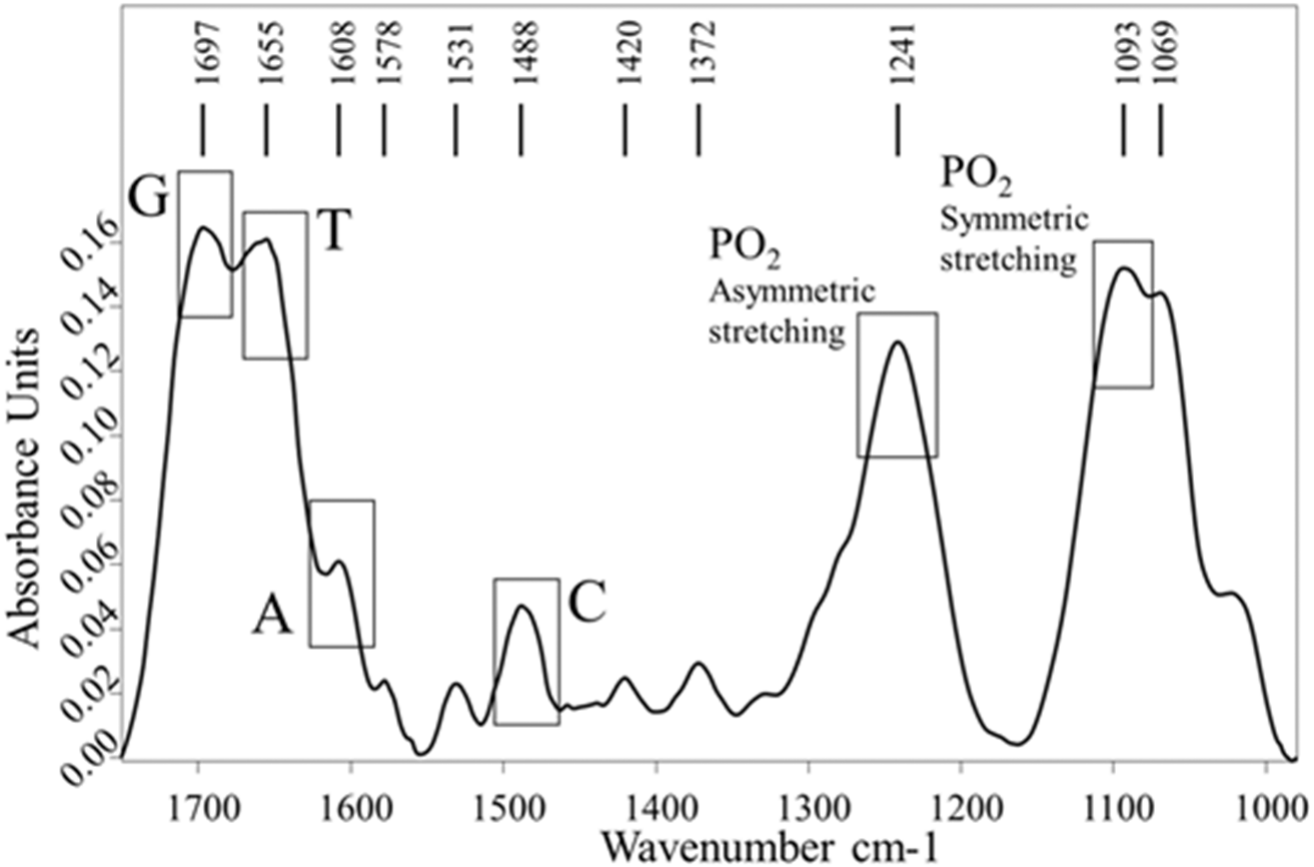
FTIR spectra of the ct-DNA. Absorptions of DNA bases (Adenine –A, Guanine –G, Cytosine –C, Thymine –T) and PO_2_ groups are marked.

The FTIR DNA spectrum was recorded to identify the specific absorption bands assigned to the DNA bases and PO_2_^−^ groups. In the FTIR spectrum of sonicated ct-DNA (Fig. 6) we identified the specific absorption bands of DNA-bases: guanine (G) at 1,697 cm^−1^, thymine (T) at 1,655 cm^−1^, adenine (A) at 1,608 cm^−1^, and cytosine (C) at 1,488 cm^−1^ (Tajmir-Riahi, N’Soukpoé-Kossi & Joly, 2009). The specific absorption bands of PO_2_^−^ groups were detected at 1,241 cm^−1^ and assigned to the symmetric stretching vibrations, and at 1,093 cm^−1^, which was assigned to the asymmetric stretching vibrations (Banyay, Sarkar & Gräslund, 2003; Cherng, 2009).

**Figure 7:**
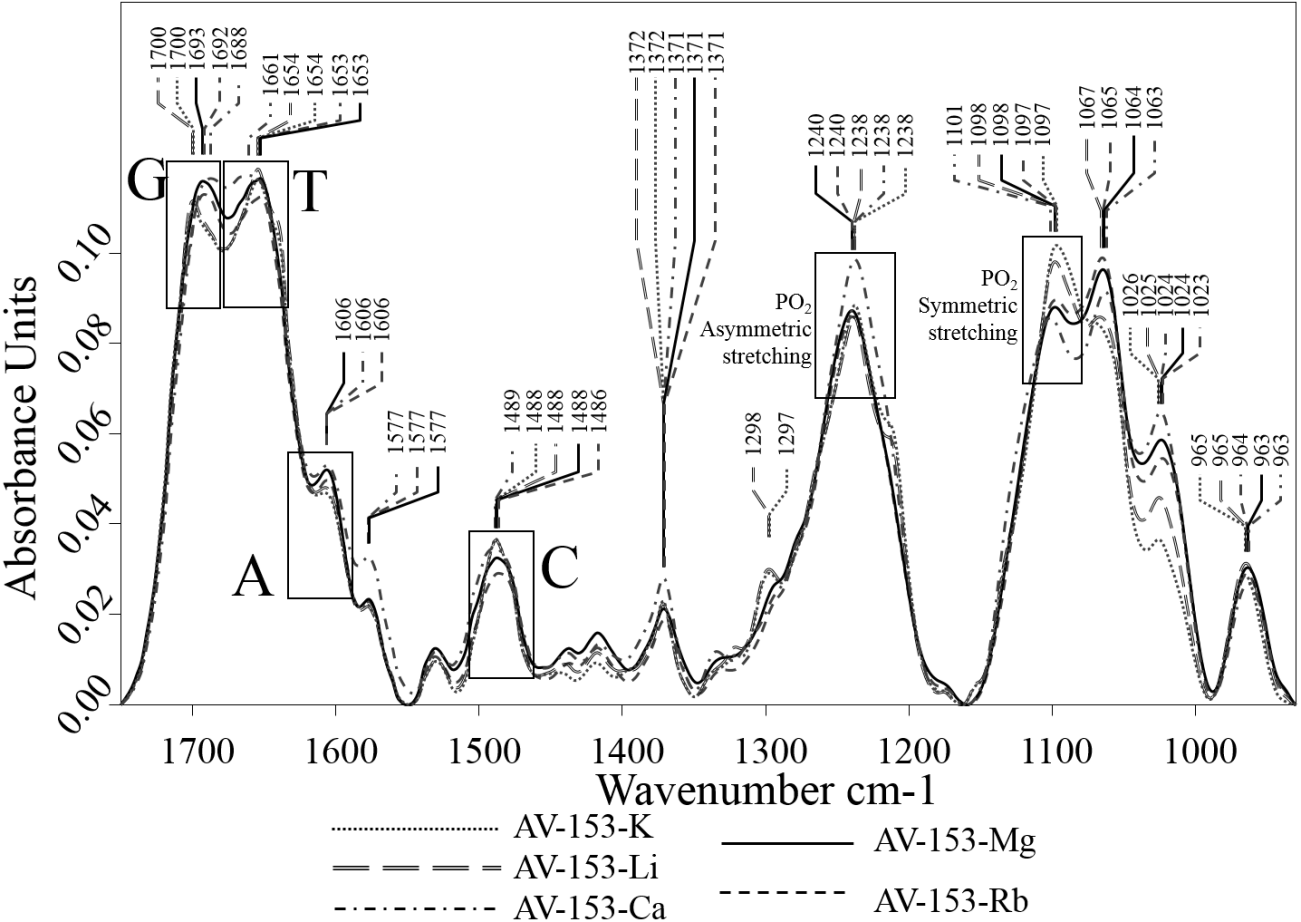
Vector normalized FTIR spectra of DNA mixed with AV-153 salts in ratio of 5:1. Wavenumber shifts and/or absorption intensity changes in all bands assigned to DNA bases are shown.

The comparison of DNA and DNA-AV-153 salt complexes spectra was used to identify the intensity and/or wavenumber shift changes that indicate the structural and conformational changes in DNA that is possibly related to interactions with AV-153 salts. The spectra of AV-153 salts mixed with DNA were vector normalized, and thus the intensity of the vibration bands is proportional to the concentration of particular bonds. The AV-153 salts spectra showed many intensive absorption bands (Fig. 5) but DNA-broad spectrum overlapped with absorption bands assigned to DNA-bases and two distinct PO_2_^−^ group vibrations bands (Fig. 6). The profile of the AV-153 salt and DNA complex spectrum, being a superposition of both components, showed all specific DNA bands, but with variable intensities and band shapes compared to that of pure DNA (Fig. 7). Spectra cross-comparison was used to find the intensity and/or frequency changes assigned to structural changes in DNA caused by the binding with the AV-153 salt. In the DNA and salt complexes spectra, the position of a specific band of G at ∼1,697 cm^−1^ was shifted to ∼1,699 cm^−1^ by Li and K; ∼1,693cm^−1^ by Mg; ∼1,692 cm^−1^ by Rb and ∼1,687 cm^−1^ by Ca. In the AV-153-Li and AV-153-K complex, the intensity of the G band decreased. The T band wavenumber (1,655 cm^−1^) was shifted by Ca to 1,661 cm^−1^, Rb to 1,653 cm^−1^ and Mg to 1,652 cm^−1^. The intensity of specific T band was affected by all AV-153 salts but most of all by Ca and Mg salts. The A band wavenumber (∼1,608 cm^−1^) was slightly shifted to 1,606 cm^−1^in the spectra of all the AV-153 salt complexes with DNA. The intensity was slightly increased by AV-153 salts of Ca, Mg and Rb. The C band intensity (1,488 cm^−1^) was not affected by AV-153-Rb, but the other salts provoked an increase in intensity and no remarkable frequency shifts were detected. The intensity of the PO_2_^−^ groups at 1,241 cm^−1^ was significantly increased by AV-153-Ca salt only. AV-153-Li produced a frequency shift from 1,241 cm^−1^ to 1,238 cm^−1^ compared to that in the DNA spectrum. All salts modified the shaped and intensity of the bands assigned to PO_2_^−^ groups at 1,093 and 1,064 cm^−1^. These PO_2_^−^ groups of DNA seem to be similarly affected by AV-153-K and -Li salts, showing two strongly overlapping bands and a decrease in intensities. Mg and Rb salts in complex with DNA significantly increased the band intensity at ∼1,065 cm^−1^ compared to that in the DNA spectrum. AV-153-Ca caused significant changes in the DNA, as a broad strongly overlapping band with maximums at ∼1,097 and ∼1,093 cm^−1^ in DNA spectra was split into two distinct bands in AV-153-Ca spectra and DNA complexes.

**Table 3:** Affinity of the AV-153-Na to bases.

Base	Binding constants of the AV-153 salts in different media		
	AV-153-Na, 5 mM Tris–HCl, 50 mM NaCl	AV-153-Na, 1 M NaCl	AV-153-Na, 8 M urea
Adenine	2.9 × 10^3^	2.3 × 10^3^	2.5 × 10^3^
Cytosine	3.6 × 10^3^	2.8 × 10^3^	3.0 × 10^3^
Guanine	8.8 × 10^3^	3.2 × 10^3^	1.9 × 10^3^
Thymine	3.5 × 10^3^	5.11 × 10^3^	3.1 × 10^3^

**Figure 8:**
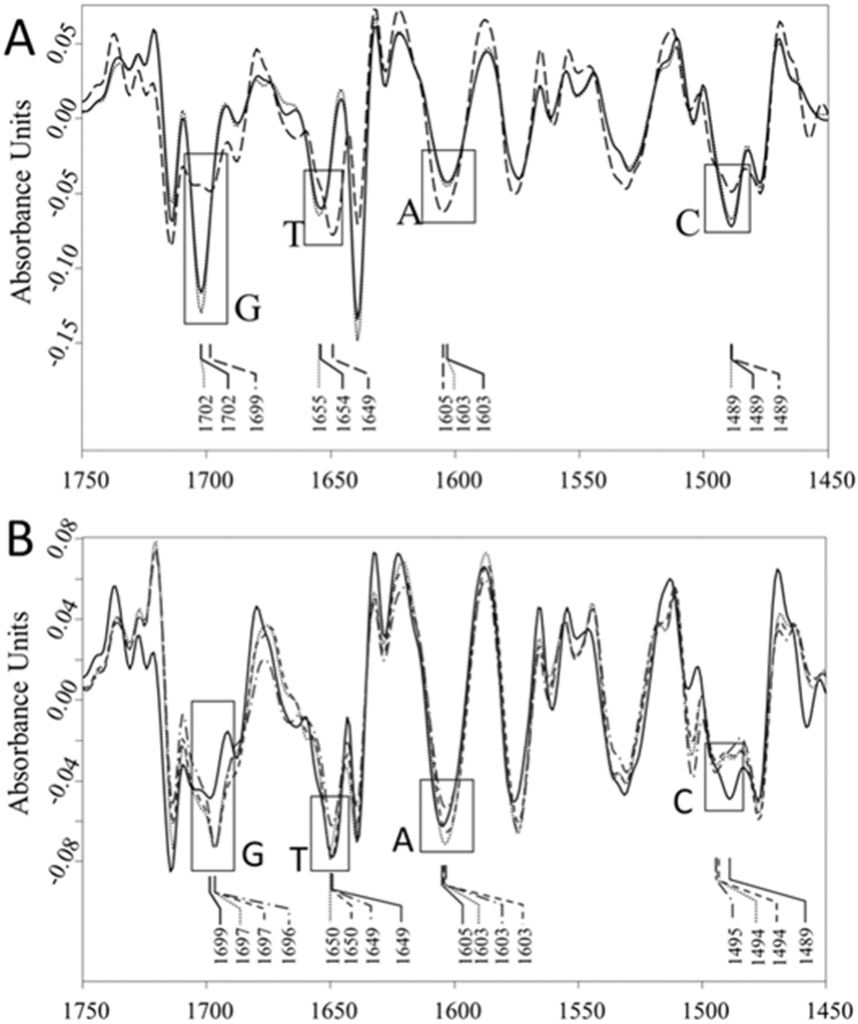
Vector normalized FTIR spectra. (A) Vector normalized second derivative spectra of the ct-DNA (solid line), dotted line –DNA with AV-153-Li (5:1), and dashed line –DNA with AV-153-K (5:1); (B) Vector normalized second derivative spectra of the ct-DNA (solid line), dotted line –DNA with AV-153-Mg (5:1), dash dotted line –DNA with AV-153-Ca (5:1), and dashed line –DNA with AV-153-Rb (5:1).

The second derivative spectra of AV-153 salts, DNA and their complexes were used to evaluate the binding of AV-153 salts with A, T, C or G. The second derivative spectra of DNA complexes with AV-153-K and AV-153-Li showed (i) significant increase of G and C absorption band intensities, (ii) a shift of T absorption band maximum from 1,649 cm^−1^ to 1,654/55 cm^−1^, (iii) a band was not significantly changed (Fig. 8A). The second derivative spectra of DNA complexes with AV-153-Ca, AV-153-Mg and AV-153-Rb showed (i) an increase in G and a decrease in C band intensities, (ii) the intensities of A and T bands were unaffected, (iii) a shift in the peak assigned to C with a maximum range of 1,489 to 1,494/95 cm^−1^ (Fig. 8B).

### Interaction with guanine quadruplexes

**Figure 9:**
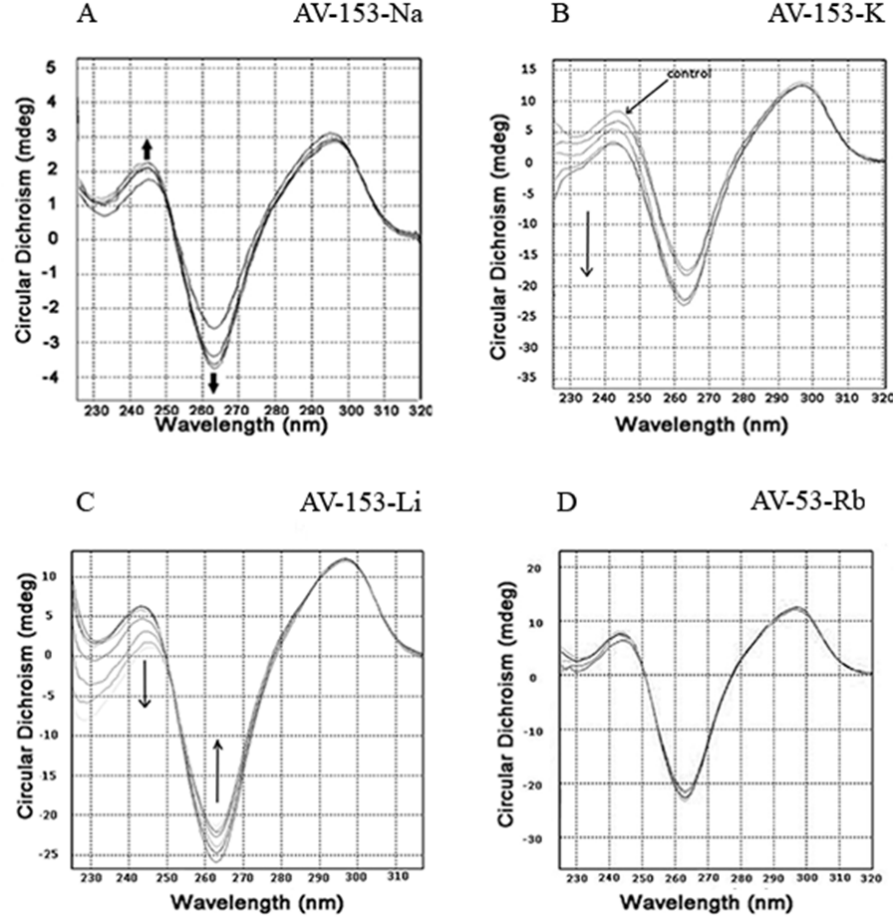
Circular dichroism spectra of the human telomere repeat oligonucleotide. Spectra were taken before and after adding of the monovalent AV-153 salts in increasing concentrations: 5 µM of the oligonucleotide were titrated in 10 mM sodium phosphate buffer, 0.3 mM EDTA, 100 mM NaCl, pH 7.2 with 10 µM of AV-153 salt at each step. (A) AV-153-Na; (B) AV-153-K; (C) AV-153-Li; (D) AV-153-Rb.

Incubation of the human telomere repeat oligonucleotide with 100 mM NaCl resulted in the formation of G4. The CD spectroscopy revealed a dominating band at 295 nm and a shallow negative band around 260 nm. Such CD spectra correspond to the two-tetrad, chair type G4. Salts from monovalent metals were tested for interaction with G4. The titration of the G4 with AV-153-Na made the peaks more pronounced (Fig. 9A), as did AV-153-K (Fig. 9B). AV-153-Li made the negative peak deeper, but the intensity of the positive peak decreased (Fig. 9C), while the AV-153-Rb did not induce any changes in the CD spectra (Fig. 9D).

**Figure 10:**
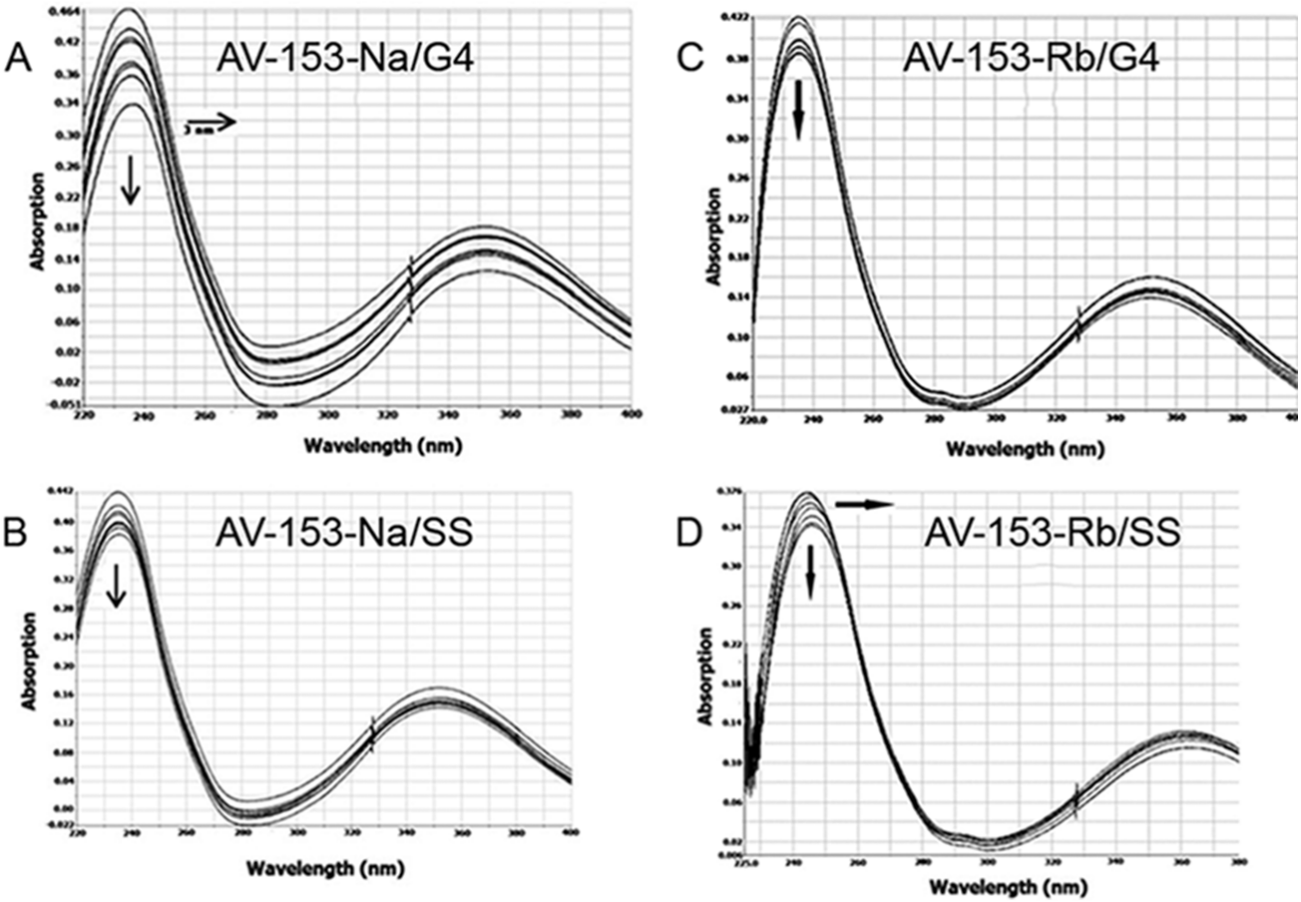
Absorption spectra of 25 µM AV-153-Na (A, B) and AV-153-Rb (C, D) in absence and presence of guanine G4 (A, C) and single-stranded (SS) human telomeric repeat (B, D). Titration was performed in 10 mM sodium phosphate buffer, 0.3 mM EDTA, 100 mM NaCl, pH 7.2, 50 nM of oligonucleotide were added each time.

Affinity to G4 and single-strand oligos was further tested using UV.VIS spectroscopy. Changes in the AV-153-Na spectra upon addition of G4 or single-strand oligonucleotides are presented in Fig. 10. Hypochromic and slight bathochromic effects are observed. Affinity to G4 is higher than to single-stranded DNA. Other salts produced similar changes in spectra (not shown), and the binding constants are given in Table 4. AV-153-Na and AV-153-K manifested higher affinity to G4, and AV-153-Li and AV-153-Rb to single-stranded DNA.

## Discussion

Metal ions modify the affinity to DNA of coordination complexes formed by organic compounds. For example, 2-[2-bromoethyliminomethyl]-4-[ethoxymethyl]phenol derivative Zn-derivative manifests a higher binding constant than copper and nickel derivatives (Lee et al., 2016). Similarly, 6-methyl-3-formyl chromone-derived hydrazine complexes with Ni have a higher binding constant to DNA than Zn and Cu complexes (Philip et al., 2017). Zn and Co phthalocyanine complexes bind to DNA in different ways; the former is an intercalator and the latter a groove binder (Özen, Günel & Baran, 2018). However, in coordination complexes, the metal does not dissociate from the organic moiety in the solution. The differences in the DNA-binding affinity of AV-153 salts revealed in this study might seem surprising if one assumes that salts should be completely dissociated in solution. However, metal ions and carbonic acid are in equilibrium during association and dissociation (Koczoń et al., 2005). All the solution measurements were performed at neutral pH, where AV-153 salts are ionized as pKs of the salts ranged between pH 4.24 and 4.52 (Leonova et al., 2019). The FTIR spectra of the compounds clearly indicate profound differences in structure also of the non-ionized forms of the salts. These might be responsible for the different effects of the compounds.

During the cyclic voltammetry experiments, the direct interaction of the compounds with DNA caused a shift in the peak potential. This reproduced the results obtained by UV/VIS spectroscopy and spectrofluorimetry (Buraka et al., 2014; Leonova et al., 2019). Moreover, these results indicate the intercalation of the compound to DNA double-helix (Sirajuddin, Ali & Badshah, 2013). We did not observe a decrease in the peak current, which is considered the main consequence of drug-DNA complex formation (Sirajuddin, Ali & Badshah, 2013). We also observed a significant increase in the peak current during the formation of the complex between DNA and terbium(III)-deferasirox (Shaghaghi et al., 2014) or 3,4-disubstituted 1,8-naphthalimide (Sharma et al., 2019). Thus, the increase in peak current, which is slight in the case of AV-153 salts, can be interpreted as a consequence of DNA and drug interaction. The ability of AV-153 salts to intercalate DNA molecule (at least partly) was confirmed by EtBr displacement assay and circular dichroism spectroscopy. The presence of a negative induced circular dichroism band, increasing with every added DNA portion with a red shift, again indicates an intercalative binding mode (Garbett, Ragazzon & Chaires, 2007; Thimmaiah et al., 2015). Interestingly, a recent study of the DNA-binding of 1,4-DHP with Ca channel blocker activity, performed mostly by electrochemical methods, also revealed an interaction of the compounds with DNA, although with much lower affinity compared to the AV-153 salts (Shahzad et al., 2019). We also compared the ability of AV-153 salts to interact with the human telomeric G4 repeat and single-stranded DNA. The presence of the affinity of some salts to the G4 opens a window for the assessment of the pharmacological properties of AV-153 salts in human telomeric-related abnormalities, while the differences in affinities show the important role of salt-forming metal ions on the molecular effect of the salts.

**Table 4:** Binding constants of the AV-153 salts with G4 forming and single-stranded human telomeric repeat.

Compound	Kb (M^−1^) G4	Kb (M^−1^) single-stranded
AV-153-Na	1.04 × 10^6^	4.49 × 10^5^
AV-153-K	4.18 × 10^5^	1.90 × 10^5^
AV-153-Li	2.23 × 10^5^	2.61 × 10^5^
AV-153-Rb	2.09 × 10^5^	2.84 × 10^5^

Cross-comparison FTIR spectra of AV-153 salts showed differences in spectrum profiles and band intensities, which reflect the differences in IR-active bonds. Although AV-153 salts spectra showed several absorption bands with similar profiles and intensities, no identical absorption bands were detected. Thus, it can be assumed that Ca, K, Mg, Li ions and Rb affect all IR-sensitive AV-153 bonds.

The FTIR spectrum of a multicomponent mixture is a result of the overlapping spectra of each constituent element. The absorption band intensity changes and/or wavenumber shifts could point to the interactions between constituents, particularly assigned to bonds specific to AV-153 salt and DNA complex. The FTIR spectra of AV-153 salts showed many strong absorption bands, evidence of their interactions with DNA, particularly with DNA-bases and PO_2_^−^ groups, should be different. After summarizing the results of FTIR spectroscopy, we concluded that: (i) the G band maximum was shifted and the intensity decreased by AV-153-K, Li and Ca salts (ii) all salts increased the intensity of T band, (iii) only AV-153-Rb did not affect the C-band, (iv) the intensity of PO_2_^−^ band at 1,241 cm^−1^, (iv) all salts changed the profile and mutual ratio of PO_2_^−^ bands at 1,097 and 1,064 cm^−1^. The FTIR spectra showed that DNA is less affected by AV-153-Rb but most of all by AV-153-Mg and AV-153-Ca.

Nafisi et al. (2008), Nafisi, Montazeri & Manouchehri (2012) and Saito et al. (2012) posit that the increase in absorption band intensities point to the partial helix destabilization of DNA, while a decrease in absorption band intensities indicate DNA stabilization, substance interaction, and conformational DNA changes.

The deconvolution (second derivative) of spectral bands is an approach for more precise evaluation of weak intensity and/or broad overlapping spectral bands. In deconvoluted spectra of DNA complexes with AV-153-K and AV-153-Li, we detected similar changes in spectral bands assigned to G, C, and T. In the spectra of the DNA complex with AV-153-Ca and AV-153-Rb, the bands assigned to G and C were modified, unlike pure DNA*.* The spectrum of the DNA complex with AV-153-Mg showed modifications of G, A, and C bands. The results of the FTIR spectroscopy showed the binding of AV-153 salts with DNA by structural and conformational changes of bonds assigned to DNA-bases and phosphate groups. Significant changes to the absorption bands assigned to the symmetric PO_2_^−^ group, stretching vibrations at ∼1,093 and ∼1,064 cm^−1^, indicate either conformational changes to the DNA molecule – form B changing to form A or Z, or partial denaturation of the molecule (Whelan et al., 2011; Wood, 2016). The characteristic IR-bands of G and C were more affected by the AV-153 salt than those of T and A. Consequently, G and C possess a higher potential to attach/bind the AV-153 salts. A UV/VIS spectroscopy of AV-153-Na complexes with bases also revealed a higher affinity to G band. The increased affinity of some AV-153 salts to the G band, revealed by the UV/VIS spectroscopy, was confirmed by FTIR spectroscopy which showed that native AV-153 salts also preferably interacted with G. The interaction with bases again indicates the possibility of an intercalative DNA-binding mode for AV-153 salts. However, interactions with phosphate groups and the different efficiency of the EtBr extrusion for different salts indicate the possibility of alternative ways of interaction, such as groove binding. The possibility of different modes of interaction with DNA was confirmed in experiments with G4 and single-stranded oligonucleotides. Hypochromic effects were observed during these experiments on the interaction of these compounds with native DNA (Buraka et al., 2014) and bases. Metal atoms again modified affinity to different oligonucleotides conformation, as some salts had a higher affinity to G4, and others to single-stranded DNA.

This study has limitations that we intend to overcome in subsequent research. The limitations concern both the methods applied and the chosen objects. NMR experiments should be performed using oligonucleotides-forming double-stranded, single-stranded, nicked- and G4-forming DNA. These oligonucleotides should be used for FTIR experiments. We also could not use acridine orange and Hoechst dyes for spectrofluorometric measurements due to the overlap of the emission spectra of these dyes with the emission spectrum of the AV-153. We hope dyes with different spectra will become available soon.

## Conclusion

Metal ions modify the affinity of AV-153 salts to DNA, DNA bases and G4 and the interaction mode between DNA and AV-153.

##  Supplemental Information

10.7717/peerj.10061/supp-1 Supplemental Information 1 Primary CD spectra

10.7717/peerj.10061/supp-2 Supplemental Information 2 Raw UV/VIS spectra

10.7717/peerj.10061/supp-3 Supplemental Information 3 Raw data and pictures for EBr displacemnt with AV-163-Li

10.7717/peerj.10061/supp-4 Supplemental Information 4 Raw data and pictures for EBr displacement with AV-153-Mg

10.7717/peerj.10061/supp-5 Supplemental Information 5 Raw data and pictures for EBr displacement with AV-153-Rb

10.7717/peerj.10061/supp-6 Supplemental Information 6 Raw data and pictures for EBr displacement with AV-153-Ca

10.7717/peerj.10061/supp-7 Supplemental Information 7 Raw data and pictures for EBr displacemnt with AV-153-K

10.7717/peerj.10061/supp-8 Supplemental Information 8 Raw data and pictures for EBr displacement with AV-153-Na

## Additional Information and Declarations

### Data Availability

The following information was supplied regarding data availability: The raw measurements are presented in Supplemental Files.
