# Supplementary figures and images for: Spectroscopic and electrochemical study of interactions between DNA and different salts of 1,4-dihydropyridine AV-153
Source: PeerJ. 2020 Nov 10;8:e10061. doi: 10.7717/peerj.10061 (PMC7664466; doi:10.7717/peerj.10061)

CD – AV-153-Ca


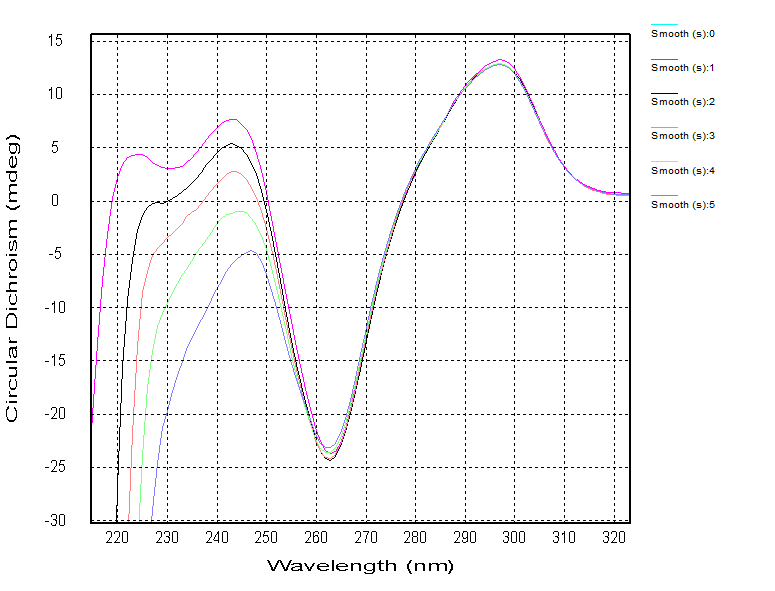


AV-153-K


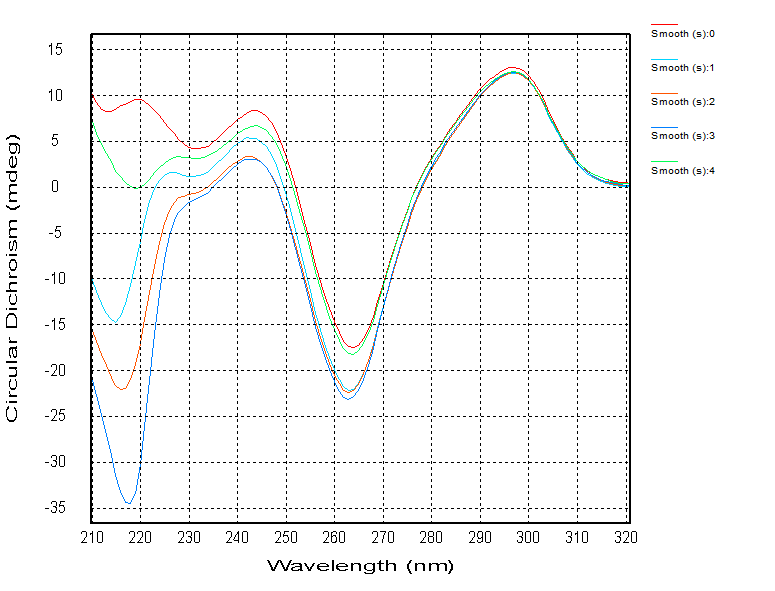


AV-153-Li


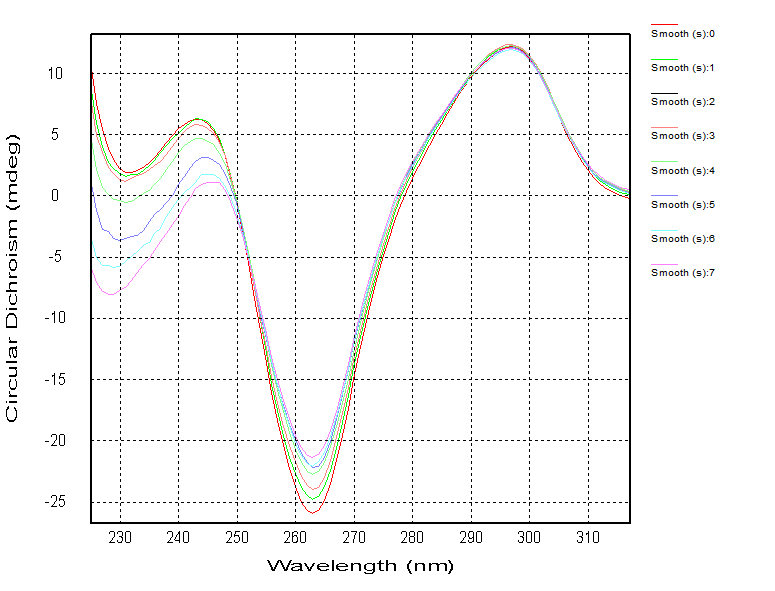


AV-153-Mg


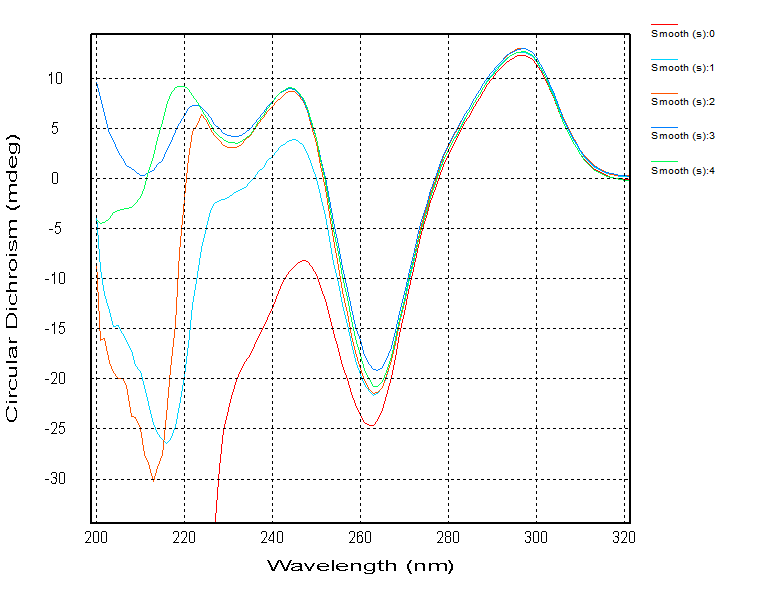


AV-153-Na


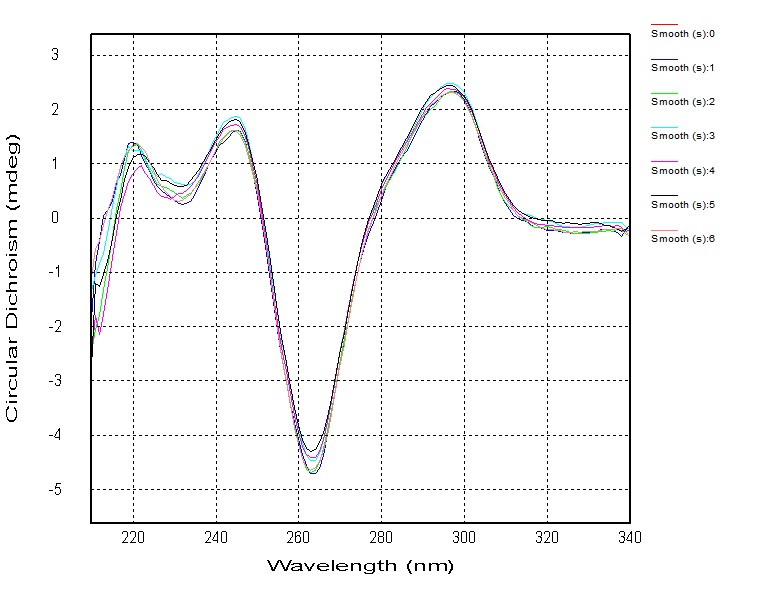


AV-153-Rb


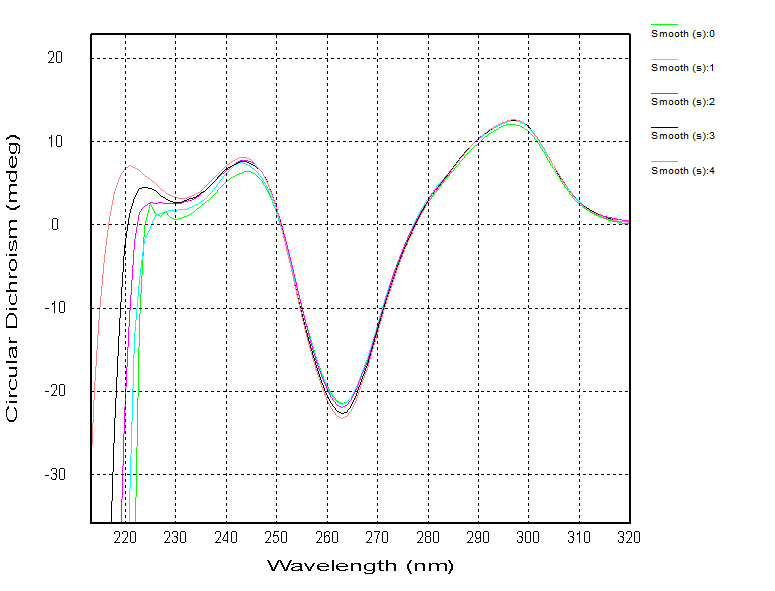

Supplement: Supplemental Information 1 [file peerj-08-10061-s001.docx]
