# Supplementary material for: Spectroscopic and electrochemical study of interactions between DNA and different salts of 1,4-dihydropyridine AV-153
Source: PeerJ. 2020 Nov 10;8:e10061. doi: 10.7717/peerj.10061 (PMC7664466; doi:10.7717/peerj.10061)
Supplement: Supplemental Information 2 [file peerj-08-10061-s002.docx]

Titration of the AV-153-K with SS oligonucleotide

Titration of the AV-153-K with G4

SS - AV-153-Li

G4 - AV-153-Li

SS-AV-153-Na

G4 – AV-153-Na

G4 – AV-153-Ca

SS-AV-153-Rb

__

G4-AV-153-Rb
